# Winning Opinion in the Voter Model: Following Your Friends’ Advice or That of Their Friends?

**DOI:** 10.3390/e27111087

**Published:** 2025-10-22

**Authors:** Francisco J. Muñoz, Juan Carlos Nuño

**Affiliations:** 1 Departamento de Matemática Aplicada, Ciencia e Ingeniería de Materiales y Tecnología Electrónica, Universidad Rey Juan Carlos, 28933 Madrid, Spain; 2 Departamento de Matemática Aplicada, Universidad Politécnica de Madrid, 28040 Madrid, Spain; juancarlos.nuno@upm.es

**Keywords:** opinion dynamics, social network, voter model, consensus time

## Abstract

We investigate a variation of the classical voter model where the set of influencing agents depends on an individual’s current opinion. The initial population is made up of a random sample of equally sized sub-populations for each state, and two types of interactions are considered: (i) direct neighbors and (ii) second neighbors (friends of direct neighbors, excluding the direct neighbors themselves). The neighborhood size, reflecting regular network connectivity, remains constant across all agents. Our findings show that varying the interaction range introduces asymmetries that affect the probability of consensus and convergence time. At low connectivity, direct neighbor interactions dominate, leading to consensus. As connectivity increases, the probability of either state reaching consensus becomes equal, reflecting symmetric dynamics. This asymmetric effect on the probability of consensus is shown to be independent of network topology in small-world and scale-free networks. Asymmetry also influences convergence time: while symmetric cases display decreasing times with increased connectivity, asymmetric cases show an almost linear increase. Unlike the probability of reaching consensus, the impact of asymmetry on convergence time depends on the network topology. The introduction of stubborn agents further magnifies these effects, especially when they favor the less dominant state, significantly lengthening the time to consensus. We conclude by discussing the implications of these findings for decision-making processes and political campaigns in human populations.

## 1. Introduction

Human behavior, especially the formation and evolution of opinions, is influenced by a complex interplay of factors, many of which remain poorly understood [1,2,3]. Among these factors, social ties play a crucial role in shaping individual opinions and preferences.

Humans inherently form networks through which information is exchanged. These networks can vary significantly depending on the context. For instance, connections formed among university peers may differ from those formed in romantic or social contexts. Nonetheless, individuals often participate in multiple overlapping networks [4,5].

The way an individual’s “neighborhood of influence” is defined can significantly affect opinion dynamics. Even when network structures are uniform, the interaction rules can determine which opinions prevail (see, for example, [6,7,8]). This study explores whether individuals benefit more by following the opinions of their immediate friends or their friends’ friends.

A personal observation inspired the question: “When I evaluate the potential success of a publication, I find a significant difference between my friend’s feedback compared with their friend’s assessments, i.e., the friends of my friend’s opinions”. Motivated by this, we investigate the dynamics of opinion formation in a population under two scenarios: (i) when the population is formed by agents that are exclusively influenced by direct friends, and (ii) when it is formed by agents influenced by their closest friend and others influenced by friends of friends. The first case will be referred to as a symmetric scenario, and the second one as an asymmetric scenario.

In this study, we simulate opinion dynamics in a population of *N* agents (nodes) on a regular ring network with circular boundary conditions. Each agent can adopt one of two states (e.g., 0 or 1). Agents in state 0 are influenced by their direct neighbors, while those in state 1 are influenced by their second neighbors (excluding direct neighbors). Using the classical voter model [9,10,11,12], with constant size and asynchronous updates, we analyze the probability of consensus and the time required to achieve it under symmetric and asymmetric neighborhood definitions.

The influence of network topology is investigated using two models: the Watts– Strogatz small-world network [13] and the Barabási–Albert scale-free network [14]. In the Watts–Strogatz model, a rewiring mechanism transforms a regular cyclic network into a random network by controlling the probability of relinking nodes, denoted by *p*. In contrast, the Barabási–Albert model generates a heterogeneous network via preferential attachment, which results in a power-law distribution of connectivity. Our results indicate that while the effect of asymmetry in the neighborhood of influence is independent of the network structure, the topology does influence the time required for the population to achieve consensus, not by altering its qualitative shape but rather in a quantitative manner.

## 2. Voter Model on a Regular Cyclic Network

We consider a population of *N* agents, each adopting one of two states (0 or 1), arranged on a regular cyclic network with connectivity *k*. Initially, the population is evenly divided between states 0 and 1, with agents randomly distributed across the network. Simulations are carried out until a final time of 1 million, guaranteeing that the population reaches one of the two consensus states, either 0 or 1. Note that the maximum connectivity that assures that the two neighborhoods, First-First (0-state) and First-Second (1-state), have no common agents and are formed by the same number of neighbors is $k_{max}=\frac{N}{2}-2$.

**Figure 1:**
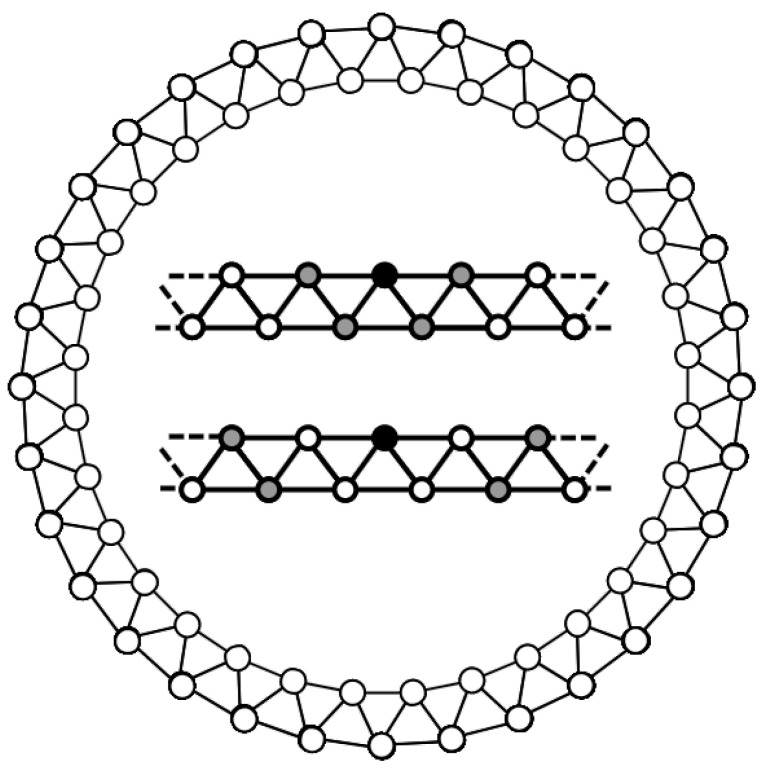
Opinion dynamics occur on a regular network as depicted in this figure. The central inset shows the different neighborhoods that are considered in this model: (Top) Nearest neighbors and (Bottom) Second neighbors (excluding the common neighbors). In this example, the network size is: N=64 and the connectivity is: k=4 (for both neighborhoods). The maximum connectivity that assures no intersection between both neighborhoods is $k_{max}=30$.

According to the classical voter model [15], at each update, a node is randomly selected, and its state is compared to that of another randomly chosen node within its neighborhood. If the two states differ, the first node adopts the second node’s state (see Figure 1). One time step is defined as *N* updates, ensuring that, on average, each node is updated once.

In the symmetric case, both states share identical neighborhoods defined by *k*-nearest neighbors, meaning all nodes are influenced equally by their closest neighbors regardless of state. In contrast, the asymmetric case introduces state-dependent neighborhoods: agents in state 0 interact with their nearest neighbors, while agents in state 1 interact with their second-nearest neighbors—skipping the immediate neighbors of the central node—yet maintaining the same neighborhood size as state 0 agents. In both scenarios, the population eventually reaches a consensus state, where all nodes adopt the same state. However, the dynamics leading to this consensus differ depending on the level of asymmetry. We analyze both the probability of reaching each consensus state (0 or 1) and the time required to reach it under symmetric and asymmetric conditions.

It should be noted that the voter model procedure is essentially a probabilistic updating rule of the cellular automaton type; that is, the probability of adopting a given state depends on the frequency of that state among a node’s neighbors. In other words, when the neighbors are in the same state, the update is deterministic and occurs with probability 1; whereas if the neighbors are in different states, the update follows a Bernoulli distribution. For k=2 in the asymmetric case, this updating rule is similar to the probabilistic rule shown in Table 1, where B(1/2) is the Bernoulli distribution with $p=\frac{1}{2}$.

In the following subsections, we present the results regarding: (i) the probability of reaching a consensus at either 0 or 1 and (ii) the time required for the population to achieve such a consensus. Then, in Section 3, we analyze these measures in the presence of a stubborn agent (also known as a zealot), and in Section 4, we explore the influence of network topology.

### 2.1. Probability of Reaching a Consensus Population

**Figure 2:**
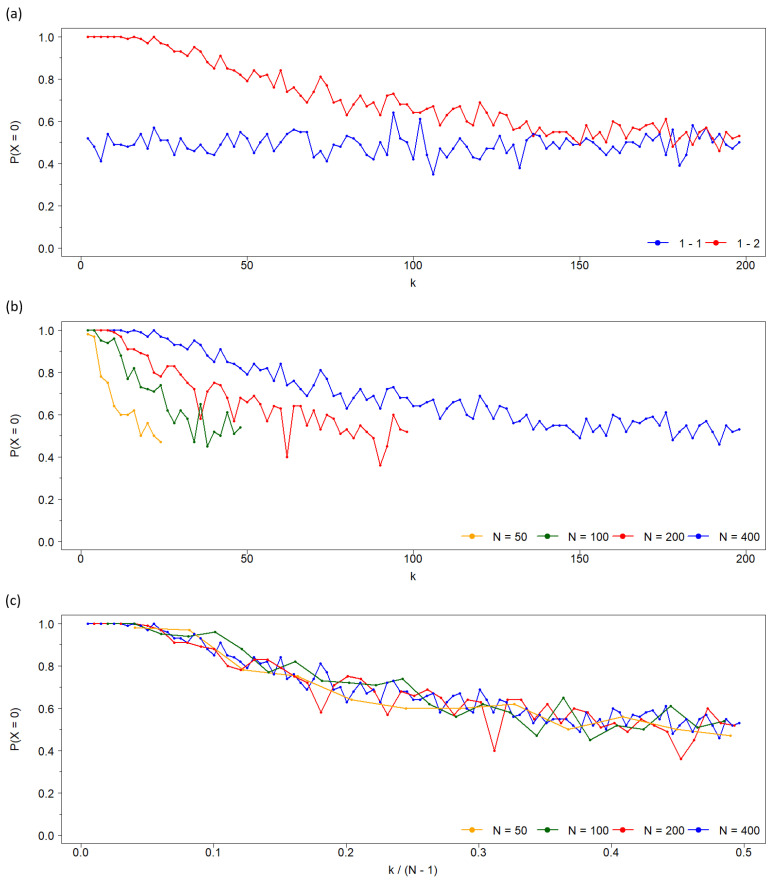
(**a**) Probability of achieving a consensus population of individuals at state 0, P(X=0), as a function of the network connectivity *k* for the two cases: (blue) symmetric population, i.e., both states have the same nearest neighborhood (First-First), and (red) asymmetric population where individuals at state 0 interact with their nearest neighbors, while individuals at state 1 interact with their second neighbors (First-Second, avoiding first neighbors). In contrast to the symmetric case, where this probability is independent of *k*, when each state has a different neighborhood, P(X=0)=1 for low values of the connectivity and decreases monotonously to 1/2 as *k* increases, approaching the symmetric dynamics. The probability is estimated from 100 simulations, and the network size is N=400. (**b**) The same probability P(X=0) in terms of *k* for four network sizes (estimated from the same number of simulations). The maximum connectivity that assures that the two neighborhoods do not intersect is $k_{max}=\frac{N}{2}-2$. (**c**) As the above panel, but normalizing the *X*-axis by the maximum connectivity N−1. As seen, the four curves converge to one, showing the asymptotic decrease to the symmetric probability P(X=0)=1/2 as *k* increases.

For symmetric neighborhoods, the probability of reaching a consensus state (0 or 1) is equal (50%) and independent of connectivity *k* [2,16]. However, in the asymmetric case, the probability depends on *k* (see Figure 2a for the probability of achieving consensus at state 0). At low *k*, consensus strongly favors state 0 (with probability 1). As *k* increases, the probability of consensus for state 1 rises, eventually reaching parity with state 0.

To understand the origin of this asymmetry, we analyze the system’s dynamics over several steps. Remarkably, after a single time step, the probabilities of reaching either state, x=0 (local neighborhood) or x=1 (nonlocal neighborhood), are equal. The asymmetry—arising from the different neighborhoods associated with each state—emerges after two steps. We focus on the case k=2 and examine the smallest interacting set that can influence the state of the central agent after two steps. In this case, this set consists of 9 nodes. We compute the probabilities of the system reaching either state, x=0 or x=1, after two time steps, considering all $2^{9}=512$ possible equiprobable initial configurations.

The following updating equation allows us to compute the state of any node $x_{i}$, for i=−2,…,2, at step n+1 as a function of the states of its neighbors, dependent on its own state, at the previous step *n*:

(1) $$\begin{array}{ccc} x_{i}\left(n+1\right) & = & F(x_{i-2}\left(n\right),x_{i-1}\left(n\right),x_{i}\left(n\right),x_{i+1}\left(n\right),x_{i+2}\left(n\right)) \end{array}$$

for i=−2,…,2 and *F* is given by:

(2) $$\begin{array}{ccc} F(x_{i-2}\left(n\right),x_{i-1}\left(n\right),x_{i}\left(n\right),x_{i+1}\left(n\right),x_{i+2}\left(n\right)) & = & \left(1-x_{i}\left(n\right)\right)(x_{i-1}\left(n\right)\;x_{i+1}\left(n\right)\\ & + & B\left(0.5\right)\;(x_{i-1}\left(n\right)+x_{i+1}\left(n\right)\\ & - & 2\;x_{i-1}\left(n\right)\;x_{i+1}\left(n\right)))\\ & + & x_{i}\left(n\right)(x_{i-2}\left(n\right)\;x_{i+2}\left(n\right)\\ & + & B\left(0.5\right)\;(x_{i-2}\left(n\right)+x_{i+2}\left(n\right)\\ & - & 2\;x_{i-2}\left(n\right)\;x_{i+2}\left(n\right))) \end{array}$$

Here $x_{-1}$ and $x_{+1}$ and $x_{-2}$ and $x_{+2}$ denote the states of the left and right first and second neighbors, respectively, and B(0.5) represents a Bernoulli distribution of p=1/2 in {0,1}. Note that the probabilistic terms arise due to the tie-breaking rule, applied when an equal number of neighbors are in each state. It is worthy to remark that other probabilities for the tie-breaking rule yield different results (work in progress).

The update equation for the state of the central node at time n+2 is:

(3) $$\begin{array}{ccc} x_{0}\left(n+2\right) & = & F(x_{-2}\left(n+1\right),x_{-1}\left(n+1\right),x_{0}\left(n+1\right),x_{+1}\left(n+1\right),x_{+2}\left(n+1\right)) \end{array}$$

Formulas (1)–(3) evaluate the probability that the central node is in state x=0 after two update steps, starting from a random initial configuration, for connectivity k=2 in the asymmetric case. Specifically, Formula (2) consists of two components: the first captures the influence of neighbors when the central node is in state x=0 (in this case, the second component becomes zero). Conversely, the second component is nonzero when the central node is in state x=1, while the first component vanishes. Each component includes two terms accounting for whether the two neighbors have the same or different states. If both neighbors share the same state, the central node adopts it; if the neighbors differ, each state has a 50% probability of being adopted by the central node. Notably, the key difference between the two components lies in the neighborhood they consider: the first depends on the immediate (nearest) neighbors, while the second depends on next-nearest neighbors, excluding the nearest ones.

When the population has symmetric neighborhoods—i.e., both state x=0 and state x=1 nodes are influenced by their nearest neighbors—the state of the central node after two update steps depends on only five nodes, including the central one. The equivalent recurrent function to Equation (2) in the symmetric case is:

(4) $$\begin{array}{ccc} F(x_{i-1}\left(n\right),x_{i}\left(n\right),x_{i+1}\left(n\right)) & = & x_{i-1}\left(n\right)\;x_{i+1}\left(n\right)\\ & + & B\left(0.5\right)\;(x_{i-1}\left(n\right)+x_{i+1}\left(n\right)-2\;x_{i-1}\left(n\right)\;x_{i+1}\left(n\right)) \end{array}$$

In contrast, when neighborhoods are state-dependent (the asymmetric case), the central node’s state after two steps depends on nine nodes from the initial configuration. In both scenarios, the update rule is applied to a set of random initial configurations, and the probability that the central node is in state x=0 after two steps is computed for both symmetric and asymmetric cases. The key finding is that this difference in how each state is adopted explains the deviations observed in this study compared to the classical Voter model.

For each initial configuration of the nine sites, we obtain the probability of the central site being in state x=0 after two steps. The results show that this probability exceeds 0.5, specifically is 0.515625. This clearly contrasts with the symmetric case, where the probability is exactly 0.5. Appendix A presents a table with the probability of achieving the state x=0 at the central site for each initial configuration, calculated from Formulas (1)–(3), for both the symmetric and asymmetric cases. As can be seen, the asymmetry observed (last row) is a consequence of the differences appearing from the 512 initial configurations.

This asymmetry explains why opinions based on local neighborhoods eventually become dominant throughout the entire population. A similar recurrent equation, although considerably longer, can be derived for the case k=4, being the probability of the central site in state x=0 after two steps estimated as 0.508174 (see Appendix B).

This tendency to equalize the probabilities of 0-consensus in both the symmetric and asymmetric cases, which we have just shown for k=2 and k=4, persists for larger values of *k*. As can be seen in Figure 2a, as the connectivity increases, the asymmetry diminishes, i.e., the probability of reaching the 0-consensus tends to $\frac{1}{2}$. This is a consequence of the dilution of local influence in opinion dynamics.

This decreasing dependence of the probability of reaching the 0-state consensus on *k* in the asymmetric case occurs independently of the network size *N* (Figure 2b). However, when plotted against normalized connectivity $\frac{k}{N-1}$, these probabilities collapse onto a single curve across different population sizes, indicating a universal dependence on the normalized parameter, as shown in Figure 2c.

**Figure 3:**
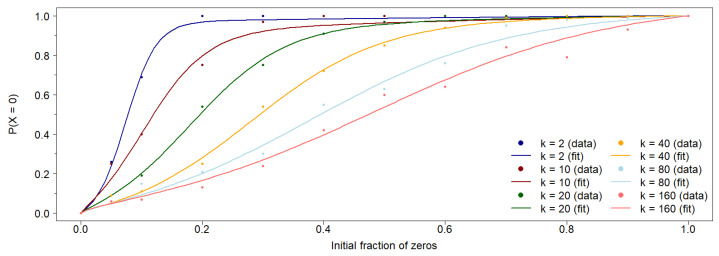
The probability of achieving 0-consensus in the population as a function of the initial fraction of agents in the 0-state, randomly distributed across the network, is shown for several values of *k* in the asymmetric case. Each data point is obtained from 100 simulations for a network with size N=400. For each value of *k*, the points can be well fitted to the Equation (5) defined in the main text, using the parameters described in Table 2.

The probability of achieving 0-consensus in the population depends on the initial conditions. As stated, the results presented above are obtained using an equally distributed initial population of agents in states 0 and 1, i.e., with a 50% fraction in state 0. Figure 3 shows the probability of achieving 0-consensus, P(X=0), as a function of the fraction of the initial population in state 0, ρ. In all cases, the initial population is randomly distributed across the vertices of the network. For each value of *k*, these probabilities can be fitted to a family of functions that pass through the points (0,0) and (1,1):

(5) $$P\left(X=0\right)=\frac{1}{1+e^{-B\;(\rho -C)}}-\frac{1}{1+e^{B\;C}}-\sqrt{\rho }\;\left(\frac{1}{1+e^{-B\;(1-C)}}-\frac{2+e^{B\;C}}{1+e^{B\;C}}\right)$$

See Table 2 for the values of parameters *B* and *C* corresponding to each *k*.

### 2.2. Consensus Time

**Figure 4:**
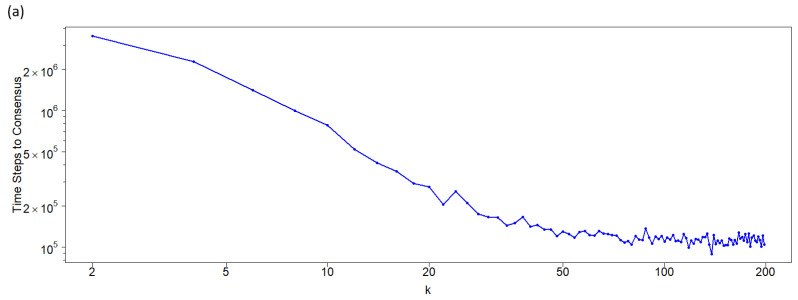

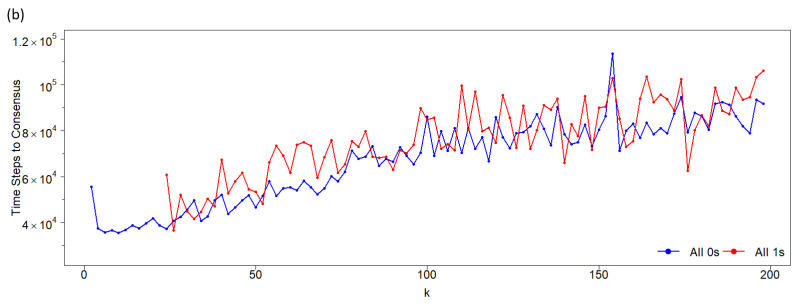
Time steps to a consensus population as a function of the network connectivity *k*. Asynchronous updating on a network formed by 400 nodes (agents). (**a**) Symmetric case: both states have the same nearest neighborhood. (**b**) Asymmetric case: nodes at state 0 interact with their nearest neighbors and nodes at state 1 interact with their second neighbors, avoiding the common neighbors. The blue curve corresponds to the 0-state consensus, and the red curve to the 1-state consensus. Note that for low *k*-values, only red points exist, since reaching the 0-state consensus has a probability of 1. Importantly, the dependence of the consensus time on *k* differs drastically between the two figures. While it decreases almost exponentially (represented by a straight line for intermediate values of *k* on a semilogarithmic scale) for the symmetric case, it increases (almost linearly) with *k* for the asymmetric case. Moreover, as can be observed, the consensus time in the asymmetric case is lower than in the symmetric one.

Figure 4 shows the results obtained from the simulations described in the previous section, where the consensus time for each state was recorded. Consensus time is defined as the first step at which the entire population adopts a single state (either 0 or 1), and it is calculated separately for each state. The reported consensus times represent the average of all simulations that result in a particular state.

For large connectivity values, the consensus times for both states are similar. However, for low connectivity values in the asymmetric case, consensus is only achieved for state 0. As shown in Figure 4a, for the symmetric case (where both states have identical neighborhoods), the consensus time decreases with increasing network connectivity, following a quasi-exponential trend. In contrast, for the asymmetric case, the consensus time increases with connectivity, as illustrated in Figure 4b.

**Figure 5:**
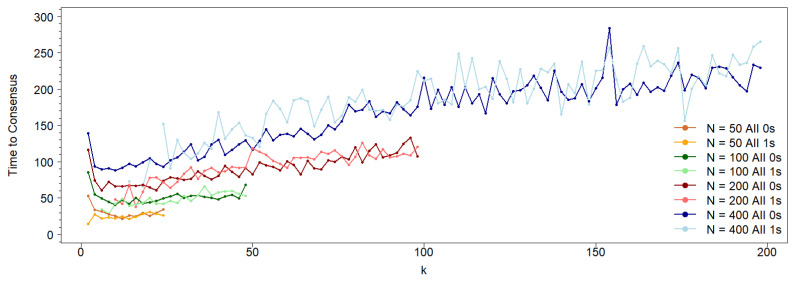
Time until consensus, i.e., time steps divided by *N*, for an initial population to reach a consensus, as a function of the network connectivity *k* for the asymmetric cases for different network sizes: 400 (blue), 200 (red), 100 (green), and 50 (orange). Light colors correspond to the time to 1-state consensus, and dark colors to 0-state consensus. Note that for low *k*-values for the four *N* sizes, only dark points appear since only consensus around the 0-state occurs. For this state, as can be observed, the consensus time exhibits a minimum value for the four sizes, at a connectivity (normalized) value that depends on *N*. The four curves can be fitted to a straight line: τ=mk+n, with the same slope *m* and the intercept n=f(N). The maximum connectivity that assures that the two neighborhoods do not intersect is $k_{max}=\frac{N}{2}-2$.

The behavior shown in Figure 4b for the asymmetric case, with a network size of N=400, is consistent across other network sizes (see Figure 5). For low *k* values, where consensus exclusively occurs in the 0-state population, the functions representing the time to consensus (measured as time steps divided by network size) increase linearly with the normalized network connectivity, $\frac{k}{N-1}$. Notably, the slope of this linear increase becomes steeper for larger network sizes, *N*.

The discrepancy between the symmetric and asymmetric cases regarding how consensus time depends on connectivity can be attributed to competition between the different neighborhood structures, which creates an asymmetry in the probabilities of reaching consensus. In the symmetric case, it is well known that higher connectivity accelerates convergence to consensus. What is surprising—and thus the value of this result—is that when two states with different neighborhoods compete, the influence of connectivity on reaching consensus is reversed. In the asymmetric case, lower connectivity results in a shorter consensus time. This is precisely due to the higher probability of reaching consensus in state 0 compared to state 1. In this scenario, there is a positive bias toward consensus in state 0, which does not occur in the symmetric case, where the probability of reaching consensus in state 0 or state 1 is the same. In the symmetric case, the population initially lacks a guiding force and remains in flux until sufficient inhomogeneity arises to steer it toward consensus.

As connectivity (*k*) increases, the probabilities of reaching consensus for states 0 and 1 converge, as does the time required to achieve consensus (Figure 4b). It is also noteworthy that for k>10, the consensus times for both states are effectively equal (see the red and blue curves in Figure 4b). For k<10, where the probability of reaching consensus in state 0 is nearly 1, the consensus time increases. This behavior is similar to the symmetric case, where both states have identical neighborhoods.

## 3. Effects of Stubborn Agents

To better understand the effect of neighborhood asymmetry on the opinion dynamics of the population, we consider the inclusion of agents whose opinions remain fixed over time, known as stubborn agents or zealots. This kind of agents can play an important role in real situations as the 2017 French presidential elections, taking part in a multistate voter model on a complex network [17]. It is well established that the presence of stubborn agents supporting both states can prevent the population from achieving consensus [18].

In the simplest case of a single stubborn agent, which represents the most unfavorable situation, the dynamics of the classical voter model with nearest neighbors are biased toward the agent’s state. For instance, if the stubborn agent permanently holds the 0-state, the entire population will eventually converge to this state, regardless of its initial configuration [19]. In essence, the stubborn agent’s state dominates and overrides the other state. The time required to achieve this consensus can also be determined in the infinite population limit [19].

**Figure 6:**
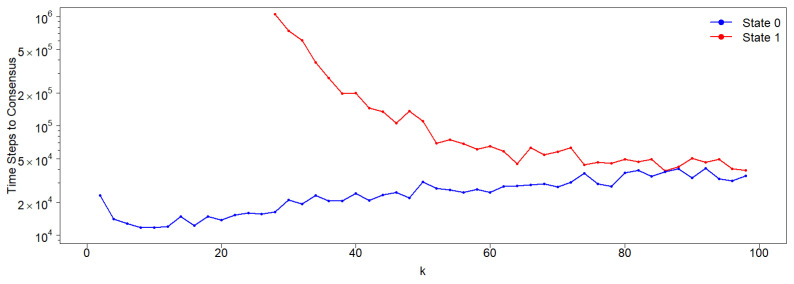
Time to consensus for both states in the presence of a stubborn agent (node), either in state 0 (blue) or 1 (red), in a network with N=200 nodes. Note that for k<28, the consensus times when the state of the stubborn agent is 1 are not depicted due to their extremely large values (larger than $10^{7}$ time steps). As in the asymmetric case without stubborn agents, the time to 1-consensus is larger than the corresponding time for the 0-consensus for all *k*-values, although these times converge as *k* increases.

In asymmetric neighborhoods, the effect of a stubborn agent remains similar to that in the symmetric case: the agent drives the population to consensus in its own state. However, the time required to reach this consensus is significantly affected by the presence of the stubborn agent (see Figure 6). If the stubborn agent holds the 0-state (nearest neighbor interactions), the consensus time is significantly reduced for any value of *k*. Conversely, if the stubborn agent holds the 1-state (second-neighbor interactions), the consensus time increases dramatically for low *k*-values. In this case, the stubborn agent effectively neutralizes the natural tendency of the population to converge to the 0-state.

## 4. Influence of Network Topology

To isolate the effect of asymmetric neighborhoods on population dynamics, we begin by considering a regular cyclic network where each node has exactly *k* first neighbors. This setup ensures that local inhomogeneities do not contribute to the asymmetric effects of neighborhoods on consensus formation. In this section, we examine how network topology influences opinion dynamics. To do so, we extend the previously defined cyclic network following the model introduced by Watts and Strogatz (1998) [13]. In this framework, the regular cyclic network corresponds to a rewiring probability of zero, while increasing the rewiring probability results in random reassignment of connections, altering key structural properties such as clustering and characteristic path length (see Figure 2 in [13]). This raises the question of whether asymmetric opinion competition affects consensus formation and if so, how.

**Figure 7:**
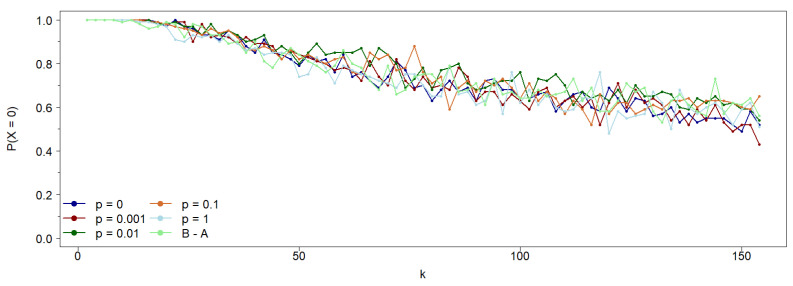
Probability of achieving a 0-consensus population as a function of the average connectivity *k* for various rewiring probabilities *p* in a cyclic Watts–Strogatz network and for the B-A scale-free network. The initial population, evenly split between states 0 and 1, is randomly distributed along N=400 sites. Notably, the consensus probability appears independent of *p*; that is, the network’s structure does not affect the final consensus state.

To address this, we replicate the simulations from Section 2 for a discrete set of rewiring probabilities, p∈[0,1]. The two extremes correspond to a regular cyclic network (p=0) and a fully random network (p=1). Figure 7 depicts the probability of achieving a 0-consensus population (i.e., a steady state where all agents adopt state 0) in an asymmetric network, where state 0 agents interact with their nearest neighbors, while state 1 agents interact with their second neighbors, avoiding the nearest ones. The key finding is that, in this type of cyclic network, randomness in connectivity does not affect the probability of reaching a 0-consensus. In other words, neighborhood asymmetry emerges for low connectivity values irrespective of the rewiring probability *p*.

**Figure 8:**
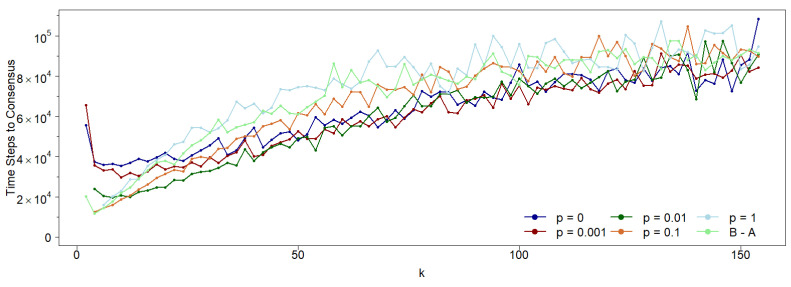
Under the same conditions as the previous figure, we show the number of time steps to reach consensus as a function of the average connectivity *k* for various *p* values. While the overall trend increases linearly with *k*, longer times to achieve a 0-consensus population are observed for higher *p* values and for the B-A scale-free network. In other words, more regular networks converge faster than either random (p=1) or inhomogeneous networks (B-A).

However, the time required to reach consensus does depend on *p*, as shown in Figure 8. Notably, for k>10, consensus time increases linearly with *k* for all values of *p*, with random networks (p=1) requiring more time to reach consensus compared to regular networks (p=0).

These results confirm that asymmetry between agent neighborhoods persists even when local neighborhoods vary, provided the network maintains a defined mean connectivity. To explore whether networks without a characteristic scale similarly influence opinion dynamics under asymmetric neighborhoods, we simulate the dynamics in a classical scale-free Barabasi–Albert (B-A) network [14]. As shown in Figure 7, asymmetry in opinion dynamics remains evident even in this case: agents influenced by their first neighbors reach consensus more easily than those updating their opinions based on second neighbors, particularly at low connectivity values. However, as noted earlier, network topology does not significantly affect consensus probability when compared to cyclic Watts–Strogatz (W-S) Small-World networks with varying rewiring probabilities. That said, the time required to reach consensus does depend on topology. As illustrated in Figure 8, the consensus time in the B-A network closely resembles that of a W-S Small-World network with p=1 (the fully random case).

## 5. Discussion

We study a population in which individuals interact on a circular network according to a classical voter model, where the state of each node determines its interaction neighborhood. Specifically, nodes in state 0 interact with their *k* nearest neighbors, while nodes in state 1 interact only with the *k* neighbors of each of their *k* first neighbors (excluding common first neighbors). We show that, in contrast to the symmetric case, a randomly initialized population with an equal number of nodes in each state tends to evolve asymptotically toward a majority of nodes in state 0—that is, individuals who interact with their immediate neighbors. Furthermore, we demonstrate that the probability of reaching a consensus in which all nodes are in state 0 depends on both the connectivity *k* and the network size *N*. In the symmetric case, the probability of reaching consensus on either state is equal (1/2) and remains independent of connectivity. In contrast, in the asymmetric case, the probability of 0-consensus increases with connectivity and scales with the population size (see Figure 4).

Regarding the time required to achieve consensus, such as 0-consensus (where all agents adopt state 0), the behavior also differs. For the symmetric case, consensus time decreases with increasing network connectivity (*k*). However, in the asymmetric case, consensus time increases with *k*, exhibiting a minimum when both states are considered equally likely to achieve consensus (see Figure 5).

This asymmetry is particularly pronounced when a stubborn agent is included in the population. If the stubborn agent holds state 0 and interacts with its nearest neighbors, the consensus time is significantly reduced. Conversely, if the stubborn agent permanently holds state 1, with a neighborhood consisting of the friends of its closest friends (excluding the latter), the time to achieve consensus in state 1 increases dramatically for low *k*-values (see Figure 6).

To explain this symmetry breaking, we solve the recursive equation (Equation (2)) after two time steps for the case k=2, finding that the probability of a node being in state 0 exceeds one half—unlike in the symmetric case. It is important to note that this asymmetry arises from the specific contagion rule used. For example, if the update rule is modified in tie situations—when a node has an equal number of neighbors in each state—different probabilities of reaching 0-consensus emerge in the asymmetric case: (i) if the node retains its current state, the probability of reaching 0-consensus is 0.5, and (ii) if the node switches its state, the probability drops below 0.5, in contrast to the behavior of the classical voter model (see Figure 2).

These results demonstrate that incorporating an asymmetric neighborhood significantly alters the opinion dynamics of the population, leading to several reflections on potential applications of this model, some of which are explored in the next section.

## 6. Concluding Remarks

The model and results presented in this paper could be particularly relevant in situations where contrasting opinions from different sources determine the final outcome. A familiar example, as described in the introduction, involves the advice provided by close friends compared to that of less familiar individuals. Close friends often tend to be more polite or hesitant to offer critical feedback, whereas friends-of-friends may provide more honest, albeit less personalized, opinions. This dynamic can sometimes lead to misleading decisions.

In a broader context, this model can help understand how misinformation or highly polarized opinions spread through direct, peer-to-peer connections (first-degree neighbors), while fact-checking or corrective information may rely on more extended networks (second-degree neighbors or more distant sources). Misinformation tends to thrive within immediate social circles, reinforcing echo chambers, as individuals often prioritize interactions within their close-knit networks. Conversely, people seeking accurate information or corrections may need to rely on second-degree connections, such as fact-checkers or experts, who lie outside their immediate peer group. In this context, the model offers insights into the persistence of polarized views and explains why corrective information often takes longer to propagate through social networks.

On a similar note, the model could also provide valuable insights into political campaign strategies. It could be used to study how certain ideological groups primarily interact within their close communities (first-degree neighbors), while second-degree connections, such as political analysts or external media outlets, may more influence others. The findings from this study could guide campaigners in effectively targeting specific groups by adopting tailored outreach strategies. For example, campaigns could focus on leveraging first-degree influencers for tight-knit communities or second-degree influencers for more diffuse audiences, depending on the nature of the opinion being addressed.

## Figures and Tables

**Table 1:** The voter model is equivalent to a probabilistic rule of the cellular automaton type. This table shows the probabilistic rule for the asymmetric case with connectivity k=2. The underscores in the situations where the central node is 1 represent the sites occupied by the nearest neighbors. When the neighbors are in different states and a tie occurs, the update of the central node is determined probabilistically according to a Bernoulli distribution with p=0.5.

**Configuration**	1_**1**_1	1_**1**_0	1**0**1	0_**1**_1	1**0**0	0_**1**_0	0**0**1	0**0**0
**New State**	**1**	B(1/2)	**1**	B(1/2)	B(1/2)	**0**	B(1/2)	**0**

**Table 2:** Fitted values for the parameters *B* and *C* of Equation (5) for different *k*. Note the low values of the Residual Sum-of-Squares (RSS) in all cases.

*k*	B	C	RSS
2	40.4144	0.07448	0.003055
10	19.0588	0.1025	0.01575
20	12.7847	0.1804	0.003038
40	9.3353	0.2819	0.004153
80	6.3784	0.3837	0.01538
160	5.3150	0.4897	0.02035

**Figure A1:**
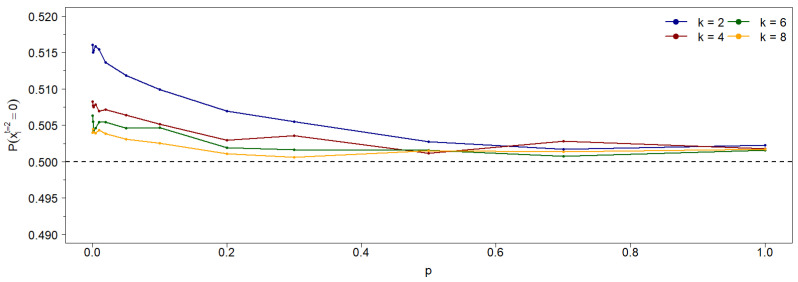
Probability of the central site reaching state 0 after two steps as a function of the rewiring probability *p* in the asymmetric case. The curves consider all possible initial state configurations of the influencing sites, for different values of *k*. Each point in the curves are obtained as an average from 1000 initial configurations. As observed, the probability of the central site reaching state 0 exceeds 0.5 for the four *k* values of the connectivity. This explain why, asymptotically, the 0-state, primarily influenced by nearest neighbor, is more likely than the 1-state, which is influenced by second neighbors (see Figure 7). Note that the points at p=0 for k=2 and k=4 provide the theoretical values stated in the main text and in this appendix, respectively.

## Data Availability

The data presented in this study are available on request from the corresponding author.

## Appendix A. Probability of Achieving 0-State at the Central Site for Each Initial Configuration After Two Steps

Table A1 shows the probability of achieving the zero state at the central site after two steps for each of the $2^{9}=512$ initial configurations. These values have been obtained from the recursive Equations (2) and (4) (see Section 2). Note that some initial configurations have the same probability of reaching the zero state in both the symmetric and asymmetric cases, while others differ. Moreover, the number of possible probability values is greater in the asymmetric case than in the symmetric one: whereas the symmetric case yields only five possible values, the asymmetric case exhibits a splitting, resulting in nine distinct values (see Table A2 and Table A3). This splitting is accompanied by an increase in the probability of the central cell reaching the zero state in the asymmetric case after two steps.

**Table A1:** Probability of achieving 0-state at the central site for each initial configuration after two steps. I.C. means Initial Configuration and $P_{11}$ and $P_{12}$ are the probabilities of achieving the 0-state after two steps in the symmetric and asymmetric case, respectively.

I.C.	$P_{11}$	$P_{12}$	I.C.	$P_{11}$	$P_{12}$	I.C.	$P_{11}$	$P_{12}$	I.C.	$P_{11}$	$P_{12}$	I.C.	$P_{11}$	$P_{12}$
000000000	1	1	001100111	1/2	3/4	011001110	1/2	3/4	100110101	1/4	3/8	110011100	1/4	5/8
000000001	1	1	001101000	3/4	3/4	011001111	1/2	5/8	100110110	1/4	1/2	110011101	1/4	1/2
000000010	1	1	001101001	3/4	3/4	011010000	1/4	1/2	100110111	1/4	3/8	110011110	1/4	1/2
000000011	1	1	001101010	3/4	1/2	011010001	1/4	1/2	100111000	1/2	1/2	110011111	1/4	3/8
000000100	3/4	3/4	001101011	3/4	1/2	011010010	1/4	3/8	100111001	1/2	1/2	110100000	1	5/8
000000101	3/4	3/4	001101100	1/2	1	011010011	1/4	3/8	100111010	1/2	1/4	110100001	1	5/8
000000110	3/4	3/4	001101101	1/2	3/4	011010100	0	1/2	100111011	1/2	1/4	110100010	1	1/2
000000111	3/4	3/4	001101110	1/2	1	011010101	0	1/4	100111100	1/4	1/2	110100011	1	1/2
000001000	1	7/8	001101111	1/2	3/4	011010110	0	1/2	100111101	1/4	3/8	110100100	3/4	1/2
000001001	1	7/8	001110000	1/4	3/4	011010111	0	1/4	100111110	1/4	3/8	110100101	3/4	3/8
000001010	1	5/8	001110001	1/4	3/4	011011000	1/4	1/2	100111111	1/4	1/4	110100110	3/4	1/2
000001011	1	5/8	001110010	1/4	5/8	011011001	1/4	1/2	101000000	3/4	3/4	110100111	3/4	3/8
000001100	3/4	1	001110011	1/4	5/8	011011010	1/4	1/4	101000001	3/4	3/4	110101000	1	1/4
000001101	3/4	7/8	001110100	0	1/2	011011011	1/4	1/4	101000010	3/4	3/4	110101001	1	1/4
000001110	3/4	7/8	001110101	0	1/4	011011100	0	1/2	101000011	3/4	3/4	110101010	1	0
000001111	3/4	3/4	001110110	0	1/2	011011101	0	1/4	101000100	1/2	1/2	110101011	1	0
000010000	1/2	1/2	001110111	0	1/4	011011110	0	1/2	101000101	1/2	1/2	110101100	3/4	1/2
000010001	1/2	1/2	001111000	1/4	1/2	011011111	0	1/4	101000110	1/2	1/2	110101101	3/4	1/4
000010010	1/2	1/2	001111001	1/4	1/2	011100000	3/4	7/8	101000111	1/2	1/2	110101110	3/4	1/2
000010011	1/2	1/2	001111010	1/4	1/4	011100001	3/4	7/8	101001000	3/4	5/8	110101111	3/4	1/4
000010100	1/4	1/2	001111011	1/4	1/4	011100010	3/4	3/4	101001001	3/4	5/8	110110000	1/2	1/2
000010101	1/4	3/8	001111100	0	1/2	011100011	3/4	3/4	101001010	3/4	3/8	110110001	1/2	1/2
000010110	1/4	1/2	001111101	0	1/4	011100100	1/2	3/4	101001011	3/4	3/8	110110010	1/2	1/2
000010111	1/4	3/8	001111110	0	1/2	011100101	1/2	5/8	101001100	1/2	3/4	110110011	1/2	1/2
000011000	1/2	3/4	001111111	0	1/4	011100110	1/2	3/4	101001101	1/2	5/8	110110100	1/4	1/4
000011001	1/2	3/4	010000000	1	1	011100111	1/2	5/8	101001110	1/2	5/8	110110101	1/4	1/8
000011010	1/2	1/2	010000001	1	1	011101000	3/4	3/4	101001111	1/2	1/2	110110110	1/4	1/4
000011011	1/2	1/2	010000010	1	1	011101001	3/4	3/4	101010000	1/4	3/8	110110111	1/4	1/8
000011100	1/4	3/4	010000011	1	1	011101010	3/4	1/2	101010001	1/4	3/8	110111000	1/2	1/4
000011101	1/4	5/8	010000100	3/4	3/4	011101011	3/4	1/2	101010010	1/4	1/4	110111001	1/2	1/4
000011110	1/4	5/8	010000101	3/4	3/4	011101100	1/2	1	101010011	1/4	1/4	110111010	1/2	0
000011111	1/4	1/2	010000110	3/4	3/4	011101101	1/2	3/4	101010100	0	1/4	110111011	1/2	0
000100000	1	7/8	010000111	3/4	3/4	011101110	1/2	1	101010101	0	0	110111100	1/4	1/4
000100001	1	7/8	010001000	1	3/4	011101111	1/2	3/4	101010110	0	1/4	110111101	1/4	1/8
000100010	1	3/4	010001001	1	3/4	011110000	1/4	5/8	101010111	0	0	110111110	1/4	1/8
000100011	1	3/4	010001010	1	1/2	011110001	1/4	5/8	101011000	1/4	3/8	110111111	1/4	0
000100100	3/4	3/4	010001011	1	1/2	011110010	1/4	1/2	101011001	1/4	3/8	111000000	3/4	3/4
000100101	3/4	5/8	010001100	3/4	7/8	011110011	1/4	1/2	101011010	1/4	1/8	111000001	3/4	3/4
000100110	3/4	3/4	010001101	3/4	3/4	011110100	0	1/2	101011011	1/4	1/8	111000010	3/4	3/4
000100111	3/4	5/8	010001110	3/4	3/4	011110101	0	1/4	101011100	0	1/4	111000011	3/4	3/4
000101000	1	1/2	010001111	3/4	5/8	011110110	0	1/2	101011101	0	0	111000100	1/2	1/2
000101001	1	1/2	010010000	1/2	1/2	011110111	0	1/4	101011110	0	1/4	111000101	1/2	1/2
000101010	1	1/4	010010001	1/2	1/2	011111000	1/4	3/8	101011111	0	0	111000110	1/2	1/2
000101011	1	1/4	010010010	1/2	1/2	011111001	1/4	3/8	101100000	3/4	7/8	111000111	1/2	1/2
000101100	3/4	3/4	010010011	1/2	1/2	011111010	1/4	1/8	101100001	3/4	7/8	111001000	3/4	5/8
000101101	3/4	1/2	010010100	1/4	3/8	011111011	1/4	1/8	101100010	3/4	3/4	111001001	3/4	5/8
000101110	3/4	3/4	010010101	1/4	1/4	011111100	0	1/2	101100011	3/4	3/4	111001010	3/4	3/8
000101111	3/4	1/2	010010110	1/4	3/8	011111101	0	1/4	101100100	1/2	3/4	111001011	3/4	3/8
000110000	1/2	3/4	010010111	1/4	1/4	011111110	0	1/2	101100101	1/2	5/8	111001100	1/2	3/4
000110001	1/2	3/4	010011000	1/2	3/4	011111111	0	1/4	101100110	1/2	3/4	111001101	1/2	5/8
000110010	1/2	3/4	010011001	1/2	3/4	100000000	1	1	101100111	1/2	5/8	111001110	1/2	5/8
000110011	1/2	3/4	010011010	1/2	1/2	100000001	1	1	101101000	3/4	1/2	111001111	1/2	1/2
000110100	1/4	1/2	010011011	1/2	1/2	100000010	1	1	101101001	3/4	1/2	111010000	1/4	3/8
000110101	1/4	3/8	010011100	1/4	5/8	100000011	1	1	101101010	3/4	1/4	111010001	1/4	3/8
000110110	1/4	1/2	010011101	1/4	1/2	100000100	3/4	3/4	101101011	3/4	1/4	111010010	1/4	1/4
000110111	1/4	3/8	010011110	1/4	1/2	100000101	3/4	3/4	101101100	1/2	3/4	111010011	1/4	1/4
000111000	1/2	1/2	010011111	1/4	3/8	100000110	3/4	3/4	101101101	1/2	1/2	111010100	0	1/4
000111001	1/2	1/2	010100000	1	5/8	100000111	3/4	3/4	101101110	1/2	3/4	111010101	0	0
000111010	1/2	1/4	010100001	1	5/8	100001000	1	7/8	101101111	1/2	1/2	111010110	0	1/4
000111011	1/2	1/4	010100010	1	1/2	100001001	1	7/8	101110000	1/4	5/8	111010111	0	0
000111100	1/4	1/2	010100011	1	1/2	100001010	1	5/8	101110001	1/4	5/8	111011000	1/4	3/8
000111101	1/4	3/8	010100100	3/4	1/2	100001011	1	5/8	101110010	1/4	1/2	111011001	1/4	3/8
000111110	1/4	3/8	010100101	3/4	3/8	100001100	3/4	1	101110011	1/4	1/2	111011010	1/4	1/8
000111111	1/4	1/4	010100110	3/4	1/2	100001101	3/4	7/8	101110100	0	1/4	111011011	1/4	1/8
001000000	3/4	3/4	010100111	3/4	3/8	100001110	3/4	7/8	101110101	0	0	111011100	0	1/4
001000001	3/4	3/4	010101000	1	1/4	100001111	3/4	3/4	101110110	0	1/4	111011101	0	0
001000010	3/4	3/4	010101001	1	1/4	100010000	1/2	1/2	101110111	0	0	111011110	0	1/4
001000011	3/4	3/4	010101010	1	0	100010001	1/2	1/2	101111000	1/4	3/8	111011111	0	0
001000100	1/2	1/2	010101011	1	0	100010010	1/2	1/2	101111001	1/4	3/8	111100000	3/4	3/4
001000101	1/2	1/2	010101100	3/4	1/2	100010011	1/2	1/2	101111010	1/4	1/8	111100001	3/4	3/4
001000110	1/2	1/2	010101101	3/4	1/4	100010100	1/4	1/2	101111011	1/4	1/8	111100010	3/4	5/8
001000111	1/2	1/2	010101110	3/4	1/2	100010101	1/4	3/8	101111100	0	1/4	111100011	3/4	5/8
001001000	3/4	3/4	010101111	3/4	1/4	100010110	1/4	1/2	101111101	0	0	111100100	1/2	5/8
001001001	3/4	3/4	010110000	1/2	1/2	100010111	1/4	3/8	101111110	0	1/4	111100101	1/2	1/2
001001010	3/4	1/2	010110001	1/2	1/2	100011000	1/2	3/4	101111111	0	0	111100110	1/2	5/8
001001011	3/4	1/2	010110010	1/2	1/2	100011001	1/2	3/4	110000000	1	1	111100111	1/2	1/2
001001100	1/2	7/8	010110011	1/2	1/2	100011010	1/2	1/2	110000001	1	1	111101000	3/4	1/2
001001101	1/2	3/4	010110100	1/4	1/4	100011011	1/2	1/2	110000010	1	1	111101001	3/4	1/2
001001110	1/2	3/4	010110101	1/4	1/8	100011100	1/4	3/4	110000011	1	1	111101010	3/4	1/4
001001111	1/2	5/8	010110110	1/4	1/4	100011101	1/4	5/8	110000100	3/4	3/4	111101011	3/4	1/4
001010000	1/4	1/2	010110111	1/4	1/8	100011110	1/4	5/8	110000101	3/4	3/4	111101100	1/2	3/4
001010001	1/4	1/2	010111000	1/2	1/4	100011111	1/4	1/2	110000110	3/4	3/4	111101101	1/2	1/2
001010010	1/4	3/8	010111001	1/2	1/4	100100000	1	7/8	110000111	3/4	3/4	111101110	1/2	3/4
001010011	1/4	3/8	010111010	1/2	0	100100001	1	7/8	110001000	1	3/4	111101111	1/2	1/2
001010100	0	1/2	010111011	1/2	0	100100010	1	3/4	110001001	1	3/4	111110000	1/4	1/2
001010101	0	1/4	010111100	1/4	1/4	100100011	1	3/4	110001010	1	1/2	111110001	1/4	1/2
001010110	0	1/2	010111101	1/4	1/8	100100100	3/4	3/4	110001011	1	1/2	111110010	1/4	3/8
001010111	0	1/4	010111110	1/4	1/8	100100101	3/4	5/8	110001100	3/4	7/8	111110011	1/4	3/8
001011000	1/4	1/2	010111111	1/4	0	100100110	3/4	3/4	110001101	3/4	3/4	111110100	0	1/4
001011001	1/4	1/2	011000000	3/4	3/4	100100111	3/4	5/8	110001110	3/4	3/4	111110101	0	0
001011010	1/4	1/4	011000001	3/4	3/4	100101000	1	1/2	110001111	3/4	5/8	111110110	0	1/4
001011011	1/4	1/4	011000010	3/4	3/4	100101001	1	1/2	110010000	1/2	1/2	111110111	0	0
001011100	0	1/2	011000011	3/4	3/4	100101010	1	1/4	110010001	1/2	1/2	111111000	1/4	1/4
001011101	0	1/4	011000100	1/2	1/2	100101011	1	1/4	110010010	1/2	1/2	111111001	1/4	1/4
001011110	0	1/2	011000101	1/2	1/2	100101100	3/4	3/4	110010011	1/2	1/2	111111010	1/4	0
001011111	0	1/4	011000110	1/2	1/2	100101101	3/4	1/2	110010100	1/4	3/8	111111011	1/4	0
001100000	3/4	1	011000111	1/2	1/2	100101110	3/4	3/4	110010101	1/4	1/4	111111100	0	1/4
001100001	3/4	1	011001000	3/4	3/4	100101111	3/4	1/2	110010110	1/4	3/8	111111101	0	0
001100010	3/4	7/8	011001001	3/4	3/4	100110000	1/2	3/4	110010111	1/4	1/4	111111110	0	1/4
001100011	3/4	7/8	011001010	3/4	1/2	100110001	1/2	3/4	110011000	1/2	3/4	111111111	0	0
001100100	1/2	7/8	011001011	3/4	1/2	100110010	1/2	3/4	110011001	1/2	3/4			
001100101	1/2	3/4	011001100	1/2	7/8	100110011	1/2	3/4	110011010	1/2	1/2	Total	256	264
001100110	1/2	7/8	011001101	1/2	3/4	100110100	1/4	1/2	110011011	1/2	1/2	Average	0.5	0.515625

**Table A2:** Number of initial configurations (# I. C.) for each probability of reaching the 0-state after two steps in the symmetric case. Note that the sum of # I. C. equals 512 and the total probability (weigthed sum) is 0.5 in this case.

**Probability**	0	0.25	0.5	0.75	1
**# I. C.**	64	128	128	128	64

**Table A3:** Number of initial configurations (# I. C.) for each probability of reaching the 0-state after two steps in the asymmetric case. Note that the sum of # I. C. equals 512 and the total probability (weigthed sum) is 264/512=0.515625.

**Probability**	0	0.125	0.25	0.375	0.5	0.625	0.75	0.875	1
**# I. C.**	28	16	80	44	144	44	108	24	24

## Appendix B. The Origin of the Asymmetry for *k* = 4

As it has been shown, the presence of different neighborhoods leads to asymmetric opinion dynamics in the voter model, influenced by the network’s connectivity. The winning opinion arises from the nearest neighbors, prevailing over those generated by the second-nearest neighbors, i.e., the friends of the closest friends. This asymmetry becomes more pronounced in networks with low connectivity, particularly at the minimum connectivity k=2 (see Section 2).

Similarly to the case k=2, we can calculate the probability of achieving any of the two states, x=0 (local neighborhood) and x=1 (nonlocal neighborhood), after two steps for k=4. For this connectivity, the smallest interacting set that affects the central agent state after two steps has 17 nodes, which yields $2^{17}$ possible configurations at step *n*.

To know the state of the central node two step ahead, i.e., at x(n+2), the following scheme can be applied:

(A1) $$\begin{array}{c} x_{i}\left(n+1\right)=F\left(x_{i-4}\left(n\right),x_{i-3}\left(n\right),x_{i-2}\left(n\right),x_{i-1}\left(n\right),x_{i}\left(n\right),x_{i+1}\left(n\right),x_{i+2}\left(n\right),x_{i+3}\left(n\right),x_{i+4}\left(n\right)\right) \end{array}$$

where i=−4,…,4 and the function *F* is given by the following:

(A2) $$\begin{array}{cc} \; & F(x_{i-4}\left(n\right),x_{i-3}\left(n\right),x_{i-2}\left(n\right),x_{i-1}\left(n\right),x_{i}\left(n\right),x_{i+1}\left(n\right),x_{i+2}\left(n\right),x_{i+3}\left(n\right),x_{i+4}\left(n\right))=\\ \; & \;(1-x_{i}\left(n\right))\\ \; & \;(x_{i-1}\left(n\right)\left(1-x_{i-1}\left(n\right)\right)\left(1-x_{i-2}\left(n\right)\right)\left(1-x_{i+1}\left(n\right)\right)\left(1-x_{i+2}\left(n\right)\right)\\ \; & \;+B(0.25)\times \\ \; & \;(x_{i-1}\left(n\right)\left(1-x_{i-2}\left(n\right)\right)\left(1-x_{i+1}\left(n\right)\right)\left(1-x_{i+2}\left(n\right)\right)+\\ \; & \;x_{i-2}\left(n\right)\left(1-x_{i-1}\left(n\right)\right)\left(1-x_{i+1}\left(n\right)\right)\left(1-x_{i+2}\left(n\right)\right)+\\ \; & \;x_{i+1}\left(n\right)\left(1-x_{i-1}\left(n\right)\right)\left(1-x_{i-2}\left(n\right)\right)\left(1-x_{i+2}\left(n\right)\right)+\\ \; & \;x_{i+2}\left(n\right)\left(1-x_{i-1}\left(n\right)\right)\left(1-x_{i-2}\left(n\right)\right)\left(1-x_{i+1}\left(n\right)\right))\\ \; & \;+B(0.5)\times \\ \; & \;(x_{i-1}\left(n\right)x_{i-2}\left(n\right)\left(1-x_{i+1}\left(n\right)\right)\left(1-x_{i+2}\left(n\right)\right)+\\ \; & \;x_{i-1}\left(n\right)x_{i+1}\left(n\right)\left(1-x_{i-2}\left(n\right)\right)\left(1-x_{i+2}\left(n\right)\right)+\\ \; & \;x_{i-1}\left(n\right)x_{i+2}\left(n\right)\left(1-x_{i-2}\left(n\right)\right)\left(1-x_{i+1}\left(n\right)\right)+\\ \; & \;x_{i-2}\left(n\right)x_{i+1}\left(n\right)\left(1-x_{i-1}\left(n\right)\right)\left(1-x_{i+2}\left(n\right)\right)+\\ \; & \;x_{i-2}\left(n\right)x_{i+2}\left(n\right)\left(1-x_{i-1}\left(n\right)\right)\left(1-x_{i+1}\left(n\right)\right)+\\ \; & \;x_{i+1}\left(n\right)x_{i+2}\left(n\right)\left(1-x_{i-1}\left(n\right)\right)\left(1-x_{i-2}\left(n\right)\right))\\ \; & \;+B(0.75)\times \\ \; & \;(x_{i-1}\left(n\right)x_{i-2}\left(n\right)x_{i+1}\left(n\right)\left(1-x_{i+2}\left(n\right)\right)+\\ \; & \;x_{i-1}\left(n\right)x_{i-2}\left(n\right)x_{i+2}\left(n\right)\left(1-x_{i+1}\left(n\right)\right)+\\ \; & \;x_{i-1}\left(n\right)x_{i+1}\left(n\right)x_{i+2}\left(n\right)\left(1-x_{i-2}\left(n\right)\right)+\\ \; & \;x_{i-2}\left(n\right)x_{i+1}\left(n\right)x_{i+2}\left(n\right)\left(1-x_{i-1}\left(n\right)\right))\\ \; & \;+x_{i-1}\left(n\right)x_{i-2}\left(n\right)x_{i+1}\left(n\right)x_{i+2}\left(n\right))\\ \; & \;+x_{i}\left(n\right)\\ \; & \;(x_{i-3}\left(n\right)\left(1-x_{i-3}\left(n\right)\right)\left(1-x_{i-4}\left(n\right)\right)\left(1-x_{i+3}\left(n\right)\right)\left(1-x_{i+4}\left(n\right)\right)\\ \; & \;+B(0.25)\times \\ \; & \;(x_{i-3}\left(n\right)\left(1-x_{i-4}\left(n\right)\right)\left(1-x_{i+3}\left(n\right)\right)\left(1-x_{i+4}\left(n\right)\right)+\\ \; & \;x_{i-4}\left(n\right)\left(1-x_{i-3}\left(n\right)\right)\left(1-x_{i+3}\left(n\right)\right)\left(1-x_{i+4}\left(n\right)\right)+\\ \; & \;x_{i+3}\left(n\right)\left(1-x_{i-3}\left(n\right)\right)\left(1-x_{i-4}\left(n\right)\right)\left(1-x_{i+4}\left(n\right)\right)+\\ \; & \;x_{i+4}\left(n\right)\left(1-x_{i-3}\left(n\right)\right)\left(1-x_{i-4}\left(n\right)\right)\left(1-x_{i+3}\left(n\right)\right))\\ \; & \;+B(0.5)\times \\ \; & \;(x_{i-3}\left(n\right)x_{i-4}\left(n\right)\left(1-x_{i+3}\left(n\right)\right)\left(1-x_{i+4}\left(n\right)\right)+\\ \; & \;x_{i-3}\left(n\right)x_{i+3}\left(n\right)\left(1-x_{i-4}\left(n\right)\right)\left(1-x_{i+4}\left(n\right)\right)+\\ \; & \;x_{i-3}\left(n\right)x_{i+4}\left(n\right)\left(1-x_{i-4}\left(n\right)\right)\left(1-x_{i+3}\left(n\right)\right)+\\ \; & \;x_{i-4}\left(n\right)x_{i+3}\left(n\right)\left(1-x_{i-3}\left(n\right)\right)\left(1-x_{i+4}\left(n\right)\right)+\\ \; & \;x_{i-4}\left(n\right)x_{i+4}\left(n\right)\left(1-x_{i-3}\left(n\right)\right)\left(1-x_{i+3}\left(n\right)\right)+\\ \; & \;x_{i+3}\left(n\right)x_{i+4}\left(n\right)\left(1-x_{i-3}\left(n\right)\right)\left(1-x_{i-4}\left(n\right)\right))\\ \; & \;+B(0.75)\times \\ \; & \;(x_{i-3}\left(n\right)x_{i-4}\left(n\right)x_{i+3}\left(n\right)\left(1-x_{i+4}\left(n\right)\right)+\\ \; & \;x_{i-3}\left(n\right)x_{i-4}\left(n\right)x_{i+4}\left(n\right)\left(1-x_{i+3}\left(n\right)\right)+\\ \; & \;x_{i-3}\left(n\right)x_{i+3}\left(n\right)x_{i+4}\left(n\right)\left(1-x_{i-4}\left(n\right)\right)+\\ \; & \;x_{i-4}\left(n\right)x_{i+3}\left(n\right)x_{i+4}\left(n\right)\left(1-x_{i-3}\left(n\right)\right))\\ \; & \;+x_{i-3}\left(n\right)x_{i-4}\left(n\right)x_{i+3}\left(n\right)x_{i+4}\left(n\right)) \end{array}$$

Here $x_{i\pm j}$ denote the states of the left and right first and second neighbors for each node *i* and B(0.5) represents a Bernoulli distribution of p=1/2 in {0,1}.

The state of the central node at time n+2 is then given by:

$$\begin{array}{cc} x_{0}\left(n+2\right)= & F(x_{-4}\left(n+1\right),x_{-3}\left(n+1\right),x_{-2}\left(n+1\right),x_{-1}\left(n+1\right),x_{0}\left(n+1\right),\\ & \;x_{+1}\left(n+1\right),x_{+2}\left(n+1\right),x_{+3}\left(n+1\right),x_{+4}\left(n+1\right)) \end{array}$$

For each initial configuration of the $2^{17}$ possible, we can obtain the probability of the central site being in state x=0 after two steps. As in the case k=2, the results also exceeds 0.5, approximately 0.508174, although closer to symmetry than in the case k=2.

This asymmetry is also present for positive values of *p* (rewiring probability). Figure A1 shows the probability that the central site is in state 0 after two steps, starting from all possible state combinations of the influencing sites, for several values of *k*. As seen in the figure, this probability is consistently greater than 0.5, highlighting the asymmetry toward the 0-state. This bias arises from the influence of nearest neighbors, which contrasts with the second-neighbor influence on 1-state agents. This observation helps explain the results presented in Figure 7.
